# Association between triglyceride-glucose index multiplied by waist circumference and H-type hypertension among Chinese adults

**DOI:** 10.3389/fcvm.2025.1589488

**Published:** 2025-07-10

**Authors:** Yiwei Peng, Ling Li, Minqi Li, Jie Wang, Tianyao Long, Liuyangyi Zheng, Xuan Tan, Xiuqin Hong

**Affiliations:** ^1^ Clinical Epidemiology Research Office, The First Affiliated Hospital of Hunan Normal University, Changsha, China; ^2^ Key Laboratory of Molecular Epidemiology, Hunan Normal University, Changsha, China; ^3^ Department of Epidemiology and Statistics, Hunan Normal University, Changsha, China; ^4^ Medical Department, Hunan Normal University, Changsha, China

**Keywords:** H-type hypertension, triglyceride glucose index, the waist circumference, cross-sectional study, community population

## Abstract

**Backgrounds:**

The triglyceride-glucose index combined with waist circumference (TyG-WC) has good predictive performance for cardiovascular disease, but the relationship of TyG-WC with H-type hypertension (HTH) is still unclear.

**Objects:**

To explore the association between TyG-WC and HTH, and provide theoretical basis for the prevention and treatment of HTH in the community population.

**Methods:**

The study used multi-stage cluster random sampling to collect representative samples from urban and rural populations in Hunan Province. HTH was defined as primary hypertension with homocysteine ≥15 μmol/L. Logistic regression and restricted cubic spline (RCS) models analyzed the TyG-WC index's association with HTH. Receiver operating characteristic (ROC) curve analysis compared TyG, WC, TyG-BMI, and TyG-WC in diagnosing HTH. An additive interaction analysis evaluated the synergistic effect of TyG and WC on HTH. Comparative analyses were conducted on the association between the TyG-WC index and both types of hypertension in the general population.

**Results:**

4,012 subjects were included in this study, and the prevalence of HTH was 19.77%. After adjusting for multiple confounding factors, participants with the highest quartile of TyG-WC had a higher risk of developing HTH compared to those with the lowest quartile of TyG-WC, with OR values of 3.182 (95% CI: 2.370–4.272, *P* < 0.001). RCS analysis revealed a significant correlation between TyG-WC index and HTH (overall trend *P* < 0.001). The correlation between TyG-WC and HTH still existed in subgroup analysis. ROC curve analysis showed that TyG-WC has higher predictive value for HTH compared to other variables (AUC = 0.676, 95%CI: 0.655–0.696, *P* = 0.010). Interaction analysis showed an additive effect between TyG and WC, with individuals having both high TyG and high WC exhibiting a 1.66 times higher HTH risk than those with low TyG and low WC. TyG-WC demonstrated a stronger association with HTH than with general hypertension.

**Conclusion:**

The TyG-WC index has high predictive value in identifying HTH, being a promising biomarker for HTH. Our findings provide theoretical basis for the prevention and treatment of HTH in the community population by controlling blood lipid, blood glucose, and waist circumference levels.

## 1 Introduction

Globally, the prevalence of hypertension has been steadily increasing, making it one of the most critical risk factors for ischemic heart disease, stroke, and other cardiovascular disorders (1). In 2017, 2.54 million people in China died from elevated systolic blood pressure, with disability adjusted life years exceeding 5% (2). A 2018 epidemiological survey on hypertension showed that the prevalence of hypertension among adult residents in China was 27.5% (3). Hypertension is the leading cause of incidence and death for cardiovascular disease among residents in China (4). The World Health Organization pointed out that the number of hypertensive patients worldwide increased from 650 million in 1990 to 1,300 million in 2019 (5). H-type hypertension (HTH) is a disease of primary hypertension accompanied by hyperhomocysteinemia (HHcy) (6). A prospective study in China found that patients with elevated homocysteine (Hcy) or hypertension had a risk of stroke that is 3.6 times and 8.2 times higher than those with normal blood pressure and Hcy levels, respectively. Patients with HHcy with hypertensionblood pressure had a significantly increased risk of cardiovascular and cerebrovascular diseases by 12.1 times (7). HTH has become a global concern, and we may be losing ground in prevention even in developmental countries.

Insulin resistance (IR) is closely related to all other components of metabolic syndrome, as well as elevated pro-inflammatory markers, thrombotic factors, and endothelial dysfunction, and is the basis for increased risk of cardiovascular diseases such as hypertension (8). In recent years, the triglyceride index (TyG) calculated from triglycerides (TG) and fasting blood glucose (FBG) has been favored by many researchers, because it is a very easy to obtain numerical value for evaluating the degree of IR (9). TG and FBG have been shown to be closely related to hypertension (10). A meta-analysis suggested that elevated fasting insulin concentration or IR estimated by steady-state model assessment was independently associated with increased risk of hypertension in the general population (11). A population-based cross-sectional study in China showed a significant correlation between the increase in TyG index and the risk of prehypertension and hypertension in Chinese adults (12). Waist circumference is an indicator of central obesity. The product of TyG index and waist circumference (TyG-WC), as a derivative index of TyG, has been shown to be significantly associated with the risk of cardiovascular disease (13). Previous study has shown correlations between TyG-WC and hypertension (12). And, a recent study reported a relationship of TyG with HTH in postmenopausal women (14), but there is currently a lack of relevant research on the correlation between TyG-WC and HTH in China.

The aim of this study is to investigate the potential correlation between TyG-WC and HTH, thereby establishing a theoretical foundation for developing effective prevention and treatment strategies for HTH within community populations.

## 2 Methods and materials

### 2.1 Study population

**Figure 1:**
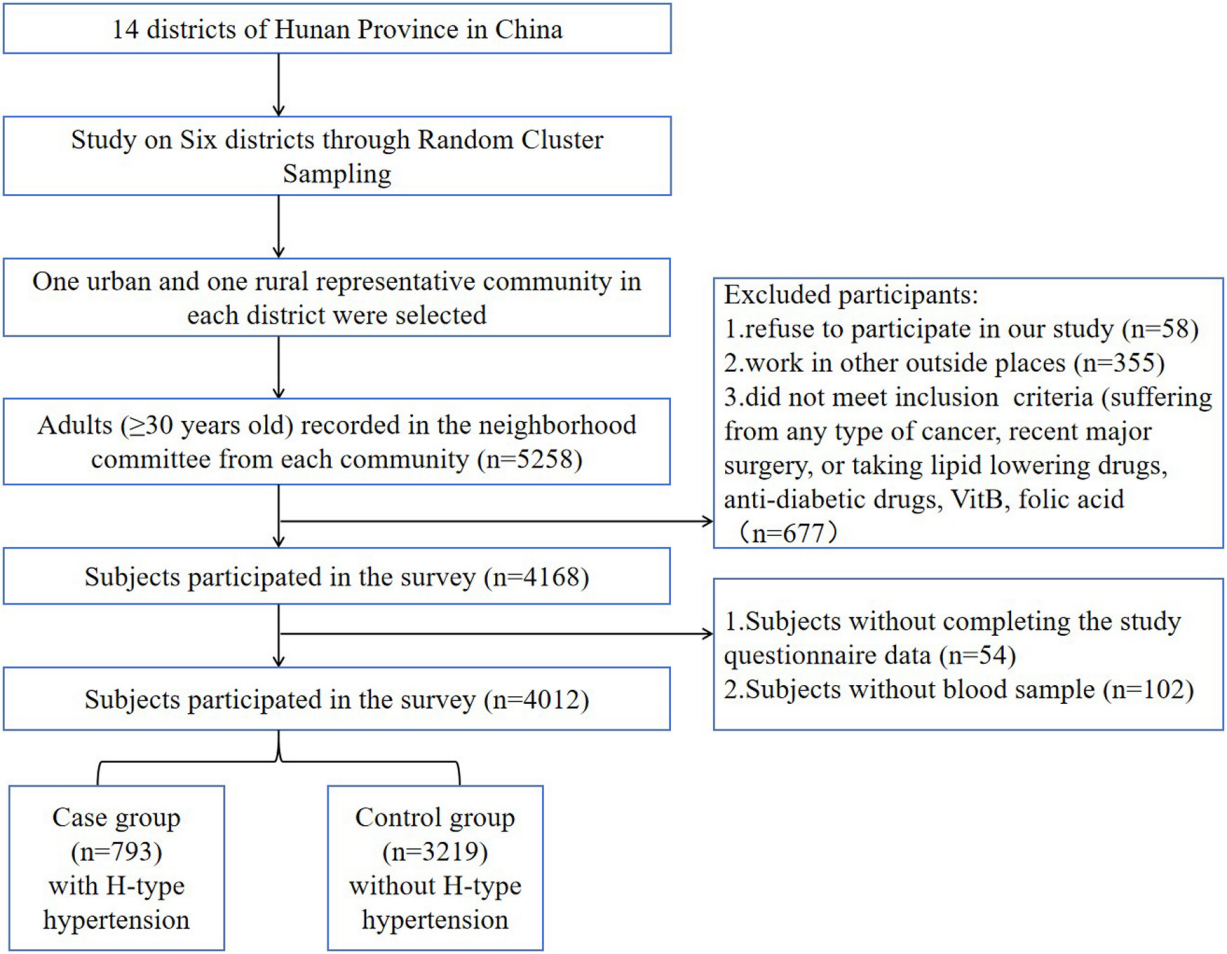
Flowchart of the sample selection.

Utilizing a multi-stage, random cluster sampling methodology, this cross-sectional study was implemented from 2013 to 2014 across six geographically and socioeconomically representative regions of Hunan Province: Changsha, Hengyang, Yongzhou, Zhuzhou, Xiangxi, and Yueyang. Within each region, two urban and two rural sampling sites were systematically selected to ensure population diversity. The study initially enrolled 5,258 community-dwelling adults, among whom 4,012 participants aged ≥30 years with complete core datasets (including survey responses, standardized physical examinations, and laboratory measurements) were ultimately included in the final analysis. In the first stage, six districts were randomly selected from the 14 districts for inclusion in the study. In the second stage, one urban and one rural community were randomly chosen from each selected district. All adults aged ≥30 years in the randomly selected communities were invited to participate. A detailed flowchart of the sampling process is presented in Figure 1. We excluded 24 subjects who had resided in the community for less than five years, had any type of cancer, or had undergone major surgery in the past six months. The randomization during the sampling process was performed by a staff member not involved in the survey, using a computer-generated random number generator. This project has been approved by the Ethics Committee of Hunan Normal University School of Medicine, and all research subjects had signed informed consent forms. Figure 1 illustrates the detailed sampling process.

### 2.2 Data collection

The study employed a standardized self-administered questionnaire to collect data through face-to-face interviews with all participants. Trained investigators, who had undergone uniform protocol training, gathered demographic information (including age and gender) and behavioral data (such as smoking history, alcohol consumption, and hypertension status). Physical examinations were conducted to measure anthropometric parameters, including height, weight, waist circumference, and blood pressure (both systolic and diastolic). Fasting blood samples were collected from participants after a minimum 8-hour fasting period to ensure standardized biochemical measurements. Blood test indicators mainly included FBG, TG, Hcy, total cholesterol (TC), high-density lipoprotein cholesterol (HDL-C), low-density lipoprotein cholesterol (LDL-C), etc.

Trained medical examiners measured height, weight, and blood pressure using standardized methods and uniform equipment. Height was measured with a stadiometer (maximum range: 2.0 m; precision: 0.1 cm), with participants required to remove shoes and hats. Weight was measured using an electronic scale (maximum capacity: 150 kg; precision: 0.1 kg), with participants wearing only light indoor clothing. Blood pressure (BP) was measured using a standard mercury sphygmomanometer (Model: A-type, Yuwell Medical Equipment & Supply Co., Ltd., Jiangsu, China) following a standardized procedure. All devices were calibrated before use (range: 0–300 mmHg; graduation: 2 mmHg). Prior to measurement, participants were instructed to: Avoid vigorous exercise, caffeine-containing beverages, and medications affecting BP for at least 1 h; Refrain from smoking for at least 15 min. Participants rested in a seated position for ≥5 min before measurement. Systolic blood pressure (SBP) and diastolic blood pressure (DBP) were recorded at the first and fifth Korotkoff sounds, respectively. Three independent BP measurements were taken, and the average value was used for data analysis.

### 2.3 Definitions and index calculation

Hypertension Diagnostic Criteria: Hypertension was defined as: SBP ≥140 mmHg and/or DBP ≥90 mmHg at measurement, or normal BP at measurement but with a history of antihypertensive medication use. Hypertension was diagnosed if either of these criteria was met. The definition of overweight/obesity is based on the criteria from *the Chinese Guidelines for the Prevention and Control of Overweight and Obesity in Adults* (15), where a BMI ≥24 kg/m^2^ is classified as overweight/ obesity. Referring to the American Heart Association standards (16), fasting serum Hcy >15 μmol/L is defined as HHcy. Hypertensive patients with Hcy concentration ≥ 15 mmol/L in their blood were defined as HTH. Smoking was defined as consuming ≥1 cigarette per day for ≥6 months (17), while alcohol drinking was defined as consuming alcohol ≥3 times per week for ≥6 months (17).

Body mass index (BMI) = weight (kg)/height (m)^2^. TyG index = ln [FPG (mg/dl) × TG (mg/dl)/2]. TyG-WC and TyG-BMI were performanced as TyG index × WC and TyG index × BMI, respectively.

### 2.4 Statistical analysis

According to the quartiles of TyG-WC (Q1, Q2, Q3, Q4) and whether the population has HTH, the baseline characteristics of the analyzed population will be grouped. Categorical variables were expressed in terms of quantity (percentage). For normally distributed metric data, mean ± standard deviation was used, for abnormally distributed metric data, median (IQR) was used. The differences in continuous variables between groups were compared using independent sample *t*-test and analysis of variance. The differences in categorical variables between groups were tested using the chi square test.

Binary logistic regression was employed to assess the association between TyG-WC and HTH. The dose-response relationship between TyG-WC and hypertension risk was visualizedusing restricted cubic spline (RCS) plots. To evaluate the diagnostic performance, receiver operating characteristic (ROC) curves were constructed and compared for TyG, WC, TyG-BMI, and TyG-WC. The area under the curve (AUC) was calculated for each parameter, and the optimal diagnostic threshold was determined based on the ROC analysis. Subgroup analyses were performed based on age, gender with corresponding interaction terms incorporated into the regression model to evaluate potential effect modifications.

To assess the interaction between TyG and WC on the risk of HTH, we calculated the additive interaction measures using Andersson et al.'s Excel-based methodology (18). The analysis included three key interaction parameters: the relative excess risk of interaction (RERI), the attributable proportion of interaction (AP), and the synergy index (SI). These measures were computed to quantify the magnitude and direction of the interaction effect between TyG and WC in relation to HTH risk. If the 95% CI of RERI and AP includes 0 or the 95% CI of SI includes 1, the additive interaction was considered not statistically significant. Using a tripartite analytical approach—comprising logistic regression analysis, RCS modeling, and ROC curve evaluation—this population-based study conducted a comprehensive comparison of TyG-WC's ability to discriminate HTH from general hypertension.

All statistical analyses were conducted using SPSS version 26.0 and R version 4.2.0. A two-sided *P* < 0.05 was considered statistically significant.

## 3 Results

### 3.1 Population characteristics

This study included 4,012 participants (mean age 54.60 ± 12.62 years; range 30–92 years; 41.0% male). Participants were stratified into HTH and non-HTH groups based on diagnostic criteria. Comparative analyses demonstrated that the HTH group showed significantly elevated levels of multiple cardiometabolic parameters compared to controls, including age, waist circumference, systolic and diastolic blood pressure, TC, TG, FBG, HDL-C, Hcy, and TyG index (all *P* < 0.05; Table 1). The baseline characteristics grouped according to the health status of the community population are shown in Supplementary Table S1.

**Table 1:** Baseline characteristics of community residents.

Characteristics	HTH	Non HTH	*X*^2^/t	*P* value
Sex, *n* (%)				
Male	444 (56)	1,200 (37.3)	92.104	<0.001
Female	349 (44)	2,019 (62.7)		
Smoking, *n* (%)				
Yes	249 (31.4)	593 (18.4)		
Current not but former smokers	69 (8.7)	168 (5.2)	87.701	<0.001
No	475 (59.9)	2 458 (76.4)		
Drinking, *n* (%)				
Yes	236 (29.8)	726 (22.6)	20.779	<0.001
No	557 (70.2)	2,493 (77.4)		
Age (years)	59.55 ± 12.69	53.38 ± 12.31	12.581	<0.001
WC (cm)	88.27 ± 10.50	83.43 ± 9.48	11.844	<0.001
SBP (mmHg)	149.15 ± 18.68	122.00 ± 17.78	38.128	<0.001
DBP (mmHg)	90.99 ± 11.40	76.83 ± 10.34	33.829	<0.001
TG (mmol·L-1)	2.51 ± 2.08	1.84 ± 1.46	8.605	<0.001
TC (mmol·L-1)	4.97 ± 1.02	4.80 ± 0.93	4.219	<0.001
Hcy (mmol·L-1)	18.27 ± 2.73	12.66 ± 8.09	32.518	<0.001
TyG (mmol·L-1)	9.14 ± 0.72	8.78 ± 0.70	13.136	<0.001
FBG (mmol·L-1)	5.99 ± 1.55	5.58 ± 1.55	6.700	<0.001
HDL-C (mmol·L-1)	1.25 ± 0.33	1.33 ± 0.33	5.802	<0.001
LDL-C (mmol·L-1)	2.58 ± 0.90	2.65 ± 0.81	1.840	0.066

When stratified by TyG index quartiles (Table 2), participants in higher quartiles exhibited progressively increasing levels of SBP, DBP, TG, TC, FBG, and HDL-C (all *P* < 0.05 for trend). Similarly, when categorized by TyG-WC quartiles (Q1:454.94–663.49; Q2:663.50–735.45; Q3:735.46–819.88; Q4:819.89–1331.60), significant positive trends were observed for all measured cardiometabolic parameters across ascending quartiles (all *P* < 0.05).

**Table 2:** Baseline information of the study population according to TyG-WC quartiles.

Characteristics	Q1 (454.94–663.49)	TyG-WC	Quartile (%)	Q4 (819.89–1,331.60)	Total	*P* value
		Q2 663.50–735.45）	Q3 (735.46–819.88）			
Sex						
Male	252 (24.0)	342 (35.7)	437 (43.6)	613 (61.1)	1 644 (41.0)	<0.001
Female	797 (76.0)	616 (64.3)	565 (56.4)	390 (38.9)	2 368 (59.0)	
Age (years)	52.02 ± 13.10	54.64 ± 12.49	56.44 ± 12.39	55.41 ± 12.04	54.60 ± 12.62	<0.001
Smoking, *n* (%)						
Yes	155 (14.8)	182 (19.0)	200 (20.0)	305 (30.4)	842 (21.0)	<0.001
No	894 (85.2)	776 (81.0)	802 (80.0)	698 (69.6)	3 170 (79.0)	
Drinking, *n* (%)						
Yes	182 (17.3)	227 (23.7)	200 (20.0)	353 (35.2)	962 (24.0)	<0.001
Never	867 (82.7)	731 (76.3)	802 (80.0)	650 (64.8)	3 050 (76.0)	
Hypertension						
Yes	215 (20.5)	327 (34.1)	418 (41.7)	578 (57.6)	1 538 (38.3)	<0.001
No	834 (79.5)	631 (65.9)	584 (58.3)	425 (42.4)	2 474 (61.7)	
WC (cm)	74.10 ± 5.34	81.25 ± 4.84	86.86 ± 5.34	95.68 ± 7.48	84.39 ± 9.88	<0.001
SBP (mmHg)	119.61 ± 19.57	125.88 ± 19.58	128.90 ± 19.21	135.38 ± 22.17	127.37 ± 20.96	<0.001
DBP (mmHg)	75.04 ± 10.43	78.51 ± 10.59	80.35 ± 11.58	84.76 ± 12.99	79.62 ± 11.97	<0.001
FBG (mmol/L)	5.12 ± 0.95	5.44 ± 1.22	5.75 ± 1.44	6.35 ± 2.10	5.66 ± 1.55	<0.001
TC (mmol/L)	4.53 ± 0.83	4.75 ± 0.84	4.88 ± 0.94	5.20 ± 1.04	4.84 ± 0.95	<0.001
TG (mmol/L)	1.08 ± 0.52	1.54 ± 0.85	2.01 ± 1.27	3.26 ± 2.29	1.97 ± 1.62	<0.001
HDL-C (mmol/L)	1.44 ± 0.37	1.35 ± 0.30	1.32 ± 0.32	1.17 ± 0.29	1.32 ± 0.33	<0.001
LDL-C (mmol/L)	2.61 ± 0.70	2.70 ± 0.76	2.68 ± 0.85	2.56 ± 0.98	2.63 ± 0.83	<0.001
Hcy (mmol/L)	12.30 ± 3.56	13.70 ± 10.10	13.92 ± 3.78	15.22 ± 10.34	13.77 ± 7.68	<0.001

### 3.2 Correlation of TyG-WC grouping and HTH

The study defined HTH as the dependent variable (coded as 1 = presence of HTH, 0 = absence of HTH), with TyG-WC grouping as the independent variable. Two analytical models were constructed: Model 1 adjusted for age and gender as covariates; Model 2 extended the adjustment by incorporating additional variables including smoking status, alcohol consumption, TG, TC, HDL-C, LDL-C, and FBG, while maintaining the adjustments for age and gender from Model 1.

**Figure 2:**
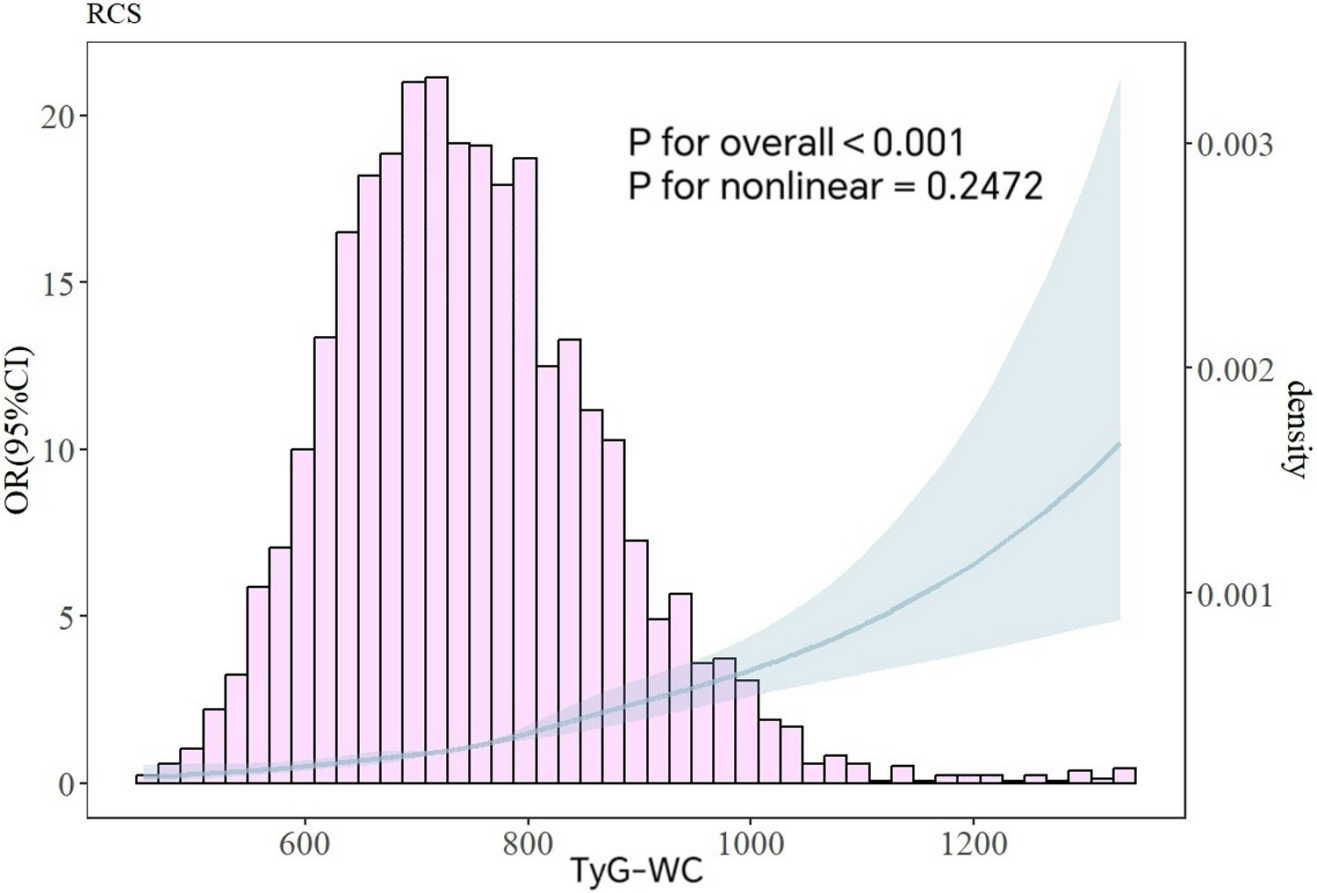
Analysis of RCS curves between TyG-WC and HTH. The variables for age, and gender were all modified.

In the unadjusted model, the highest TyG-WC quartile showed a significantly higher risk of HTH compared to the lowest TyG-WC quartile (OR = 4.988, 95% CI: 3.898–6.383). After adjusting for sex and age in Model 1, the hypertension risk in the highest TyG-WC quartile was 4.119 times that of the lowest quartile (OR = 4.119, 95% CI: 3.191–5.316). After adjusting for the covariates in Model 3, the highest TyG-WC group had a 3.182-fold increased risk of HTH compared to the lowest TyG-WC group (OR = 3.182, 95% CI: 2.370–4.272). In three gradually adjusted models, for every quarter increase in TyG-WC grouping, the incidence of HTH gradually increased, and TyG-WC remained positively correlated with HTH. As shown in Table 3. According to the RCS plot, there was a significant positive correlation between TyG-WC and the risk of HTH when it was a continuous variable (overall trend *P* < 0.001, non-linear *P* = 0.2472). As shown in Figure 2.

**Table 3:** Association between TyG-WC index and HTH in different models.

Characteristics	Model 1	*P* value	Model 2	*P* value	Model 3	*P* value
	OR (95% CI)		OR (95% CI)		OR (95% CI)	
TyG-WC	1.005 (1.004–1.006)	<0.001	1.005 (1.004–1.005)	<0.001	1.004 (1.004–1.005)	<0.001
TyG-WC (%)						
Q1	1.00		1.00		1.00	
Q2	1.652 (1.253–2.177)	<0.001	1.473 (1.112–1.953)	0.007	1.368 (1.027–1.822)	0.032
Q3	2.761 (2.135–3.570)	<0.001	2.292 (1.762–2.982)	<0.001	2.063 (1.569–2.714)	<0.001
Q4	4.988 (3.898–6.383)	<0.001	4.119 (3.191–5.316)	<0.001	3.182 (2.370–4.272)	<0.001
*P* for trend		<0.001		<0.001		<0.001

Model 1: unadjusted.

Model 2: Adjust age and gender.

Model 3: Adjust for age, gender, smoking, alcohol consumption, triglycerides, total cholesterol, high-density lipoprotein, low-density lipoprotein, and fasting blood glucose.

### 3.3 ROC curves for TyG, WC, TyG-BMI, and TyG-WC

**Figure 3:**
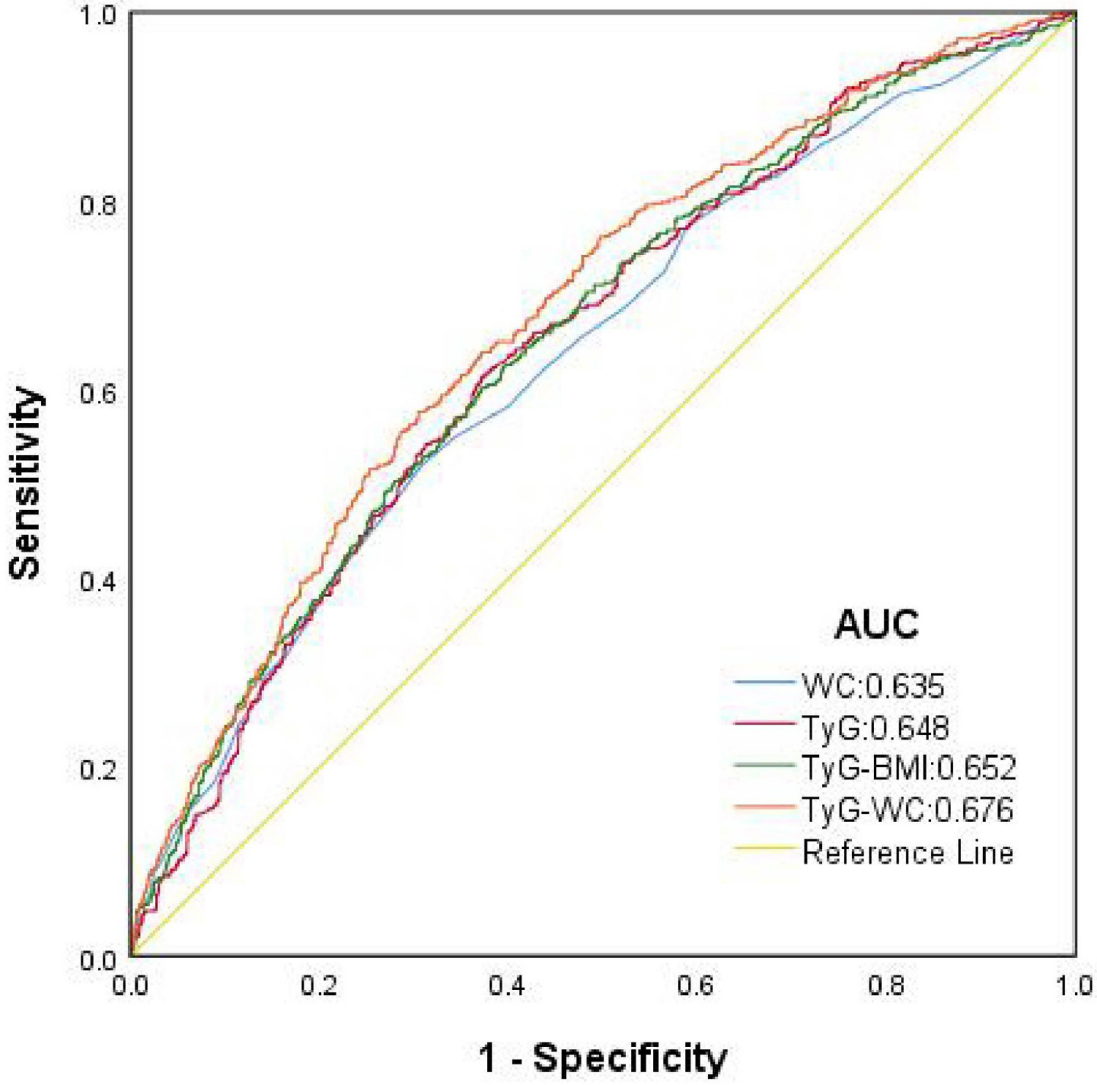
ROC curve for predicting HTH using multiple continuous variables. AUC, area under the curve; WC, waist circumference; TyG, triglyceride-glucose index; TyG-WC, triglyceride glucose-waist circumference index; TyG-BMI, triglyceride glucose- body mass index.

To evaluate the ability of TyG-WC to assess its correlation with HTH, receiver operating ROC curves were constructed and analyzed. The diagnostic performance of four variables—TyG, WC, TyG-BMI, and TyG-WC was systematically compared in identifying HTH. Among these variables, TyG-WC demonstrated superior association, as evidenced by its significantly larger area under the ROC curve (AUC = 0.676, *P* = 0.010). The optimal cutoff value for TyG-WC was determined to be 781.9640, yielding a sensitivity of 57.6% and specificity of 69.5%. These findings indicated that TyG-WC exhibited the strongest related ability among the evaluated parameters for identifying HTH. In this study, we found that the accuracy of TyG-BMI identification [AUC: 0.652 (95% CI: 0.631–0.673, *P* = 0.011), specificity 62.9%, sensitivity 60.2%] was higher than that of TyG [AUC: 0.648 (95% CI: 0.627–0.669, *P* = 0.011), specificity 62.8%, sensitivity 61.4%] and WC [AUC: 0.635 (95% CI: 0.613–0.656, *P* = 0.011), specificity 69.2%, sensitivity 52.0%], as shown in Figure 3.

### 3.4 Subgroup analysis

**Figure 4:**
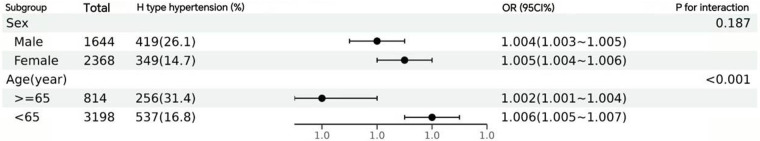
Subgroup analysis of TyG-WC and HTH.

In order to investigate the differences in the association between TyG-WC and HTH in different populations, subgroup analysis was conducted on the population based on sex and gender and relevant forest plots were drawn, namely Figure 4. The results indicated that all subgroups of TyG-WC were positively correlated with HTH (*P* < 0.001), and there was a significant interaction between age subgroups (*P* for interaction < 0.001).

### 3.5 Analysis of the interaction between TyG and WC

The impact of the interaction between TyG and WC on the risk of HTH was analyzed in Table 4. The results showed that after multivariable adjustment, TyG and WC had an additive interaction effect on the incidence of HTH patients, RERI, 95% CI: −0.635, −1.218–−0.052; AP, 95% CI: −0.383, −0.745–−0.021; SI, 95% CI: 0.509, 0.273–0.950. Compared to low TyG and low WC, the simultaneous presence of high TyG and high WC increased the risk of HTH, with an OR (95% CI) value of 1.659 (1.100–2.502). In addition, compared with high WC and low TyG, the OR of high TyG and low WC was higher (2.059, 95% CI: 1.474–2.874). As shown in Table 4.

**Table 4:** The interaction between TyG and WC.

Interactivity Type	Variable	OR	*P* value/interaction
	TyG	2.060 (1.474–2.878)	<0.001
Potentiation	WC	1.235 (0.891–1.712)	0.206
	TyG*WC	0.633 (0.452–0.885)	0.042
	low TyG low WC	1.000	<0.001 <0.001 <0.001
	low WC high TyG	2.059 (1.474–2.874)	
Additive effect	high WC low TyG	1.235 (0.890–1.714)	
	high WC high TyG	1.659 (1.100–2.502)	

Model: Adjust for age, gender, smoking, alcohol consumption, triglycerides, total cholesterol, high-density lipoprotein, low-density lipoprotein, and fasting blood glucose.

### 3.6 Comparative analysis of the association between TyG-WC and two types of hypertension

In the multivariable-adjusted regression models with sequential covariate adjustments, TyG-WC maintained a robust positive association with both hypertension subtypes (OR = 1.696, 95% CI: 1.532–1.877; OR = 1.342, 95% CI: 1.047–1.720), though the effect sizes exhibited minor fluctuations across models. Notably, this significant association persisted when TyG-WC was analyzed as a continuous variable (Supplementary Table S2).

RCS analyses demonstrated a clear linear dose-response relationship between TyG-WC levels and two types of hypertension risk. Specifically, for H-type hypertension, we observed a strong linear trend (*P* for overall trend <0.001) without significant nonlinearity (*P* for nonlinearity = 0.304). A similar linear association was evident for general hypertension (*P* for overall trend <0.001, *P* for nonlinearity = 0.3381). Importantly, comparative analysis revealed that TyG-WC exhibited a more substantial effect on H-type hypertension risk compared to general hypertension (Supplementary Figure S1). ROC curve analysis indicated superior discriminatory performance of TyG-WC in detecting HTH [AUC: 0.723 (95% CI: 0.702–0.744, *p* = 0.011), specificity 76.8%, sensitivity 57.6%] relative to general hypertension [AUC: 0.653 (95% CI: 0.629–0.677, *p* = 0.012), specificity 67.3%, sensitivity 55.1%], as evidenced by its stronger association metrics (Supplementary Figure S2).

## 4 Discussions

In this cross-sectional study based on a community population in Hunan, China, we found that a higher TyG-WC index was associated with the occurrence of H-type hypertension. In the population-based cross-sectional analysis, after comprehensive adjustment for potential confounders, TyG-WC demonstrated a robust and statistically significant positive association with HTH, both when analyzed as continuous and categorical variables. Subgroup analyses further reinforced the consistency and reliability of this relationship. ROC curve analysis revealed that TyG-WC had significantly better predictive performance for HTH compared to WC, TyG, and TyG-BMI. RCS analysis of nonlinear relationships consistently confirmed the presence of this association. Interaction analysis identified a synergistic effect between TyG and WC, with individuals exhibiting both high TyG and high WC showing a 1.659-fold increased risk of developing HTH relative to those with low TyG and low WC group. In the comparative analysis of the association between TyG-WC and the two hypertension subtypes, TyG-WC demonstrated a stronger discriminatory association with HTH than with general hypertension.

Hcy is a thiol amino acid produced by demethylation of methionine in liver, muscle, and other tissues. There are four pathways in human metabolism, and HHcy is produced when metabolism is abnormal (19). HHcy is an independent risk factor for cardiovascular and cerebrovascular diseases, which can lead to pathological changes such as endothelial damage, increasing the risk of cardiovascular and cerebrovascular diseases (20). The prevalence of hypertension among Chinese adults is 23.2%, with approximately 75% of patients having HTH (21). Domestic scholars have shown that the combined effect of Hhcy and hypertension can increase the risk ratio of vascular disease to 11.3 (22). HTH has become an important public health issue.

A cross-sectional study of Chinese men showed that FPG is significantly associated with the incidence rate of hypertension (23). A cohort study found that the increase of FPG change track was associated with a higher probability of hypertension when assessing the association between FPG change track and hypertension incidence rate in Chinese population (24). The hypertriglyceridemic waist (HTGW) phenotype is defined as an increase in waist circumference and triglyceride concentration. A cross-sectional study of Chinese adults showed that HTGW phenotype is closely related to hypertension (25). TyG index is a comprehensive statistical indicator that includes TG and FPG levels. Research has shown that due to its high sensitivity and specificity, it can serve as an effective alternative biomarker for IR (26).

Waist circumference is one of the indicators of central obesity, and an increase in waist circumference has been shown to increase the incidence of cardiovascular disease (27). Waist circumference is closely related to the occurrence and development of diseases such as blood lipids and metabolic abnormalities (28). Research has shown that WC is superior to body mass index (BMI) in predicting chronic diseases such as obesity and hypertension (29). Domestic research has shown that the association between WC and Hcy is closer than BMI, and WC can be used as a predictive indicator for HHcy and hypertension (30). WC is also one of the indicators of abdominal obesity, which has been shown to have a stronger correlation with subcutaneous fat rather than visceral adipose tissue (31). Meanwhile, it is also closely related to IR. TyG and WC have been well documented to be highly correlated with hypertension. TyG is an independent positively correlated factor for the risk of hypertension in the central obesity pre obesity group (32).

IR is associated with hypertension and is now considered a pathogenic and predictive factor for hypertension (33), particularly in HTH (34). The TyG index is considered a substitutability index for IR (35), and adding obesity indicators such as BMI and WC to the TyG index may be more accurate than using the TyG index alone (36). Recent studies have shown that the TyG index is associated with incident hypertension (37–39) and is linked to various cardiovascular diseases (40, 41).

A cross-sectional study showed that TG-WC index is the best predictor of IR (42). A cohort study (13) showed that in the pattern of changes in TyG related indicators, TyG-WC has the strongest association with the risk of cardiovascular disease. The study by Miao Huanhuan et al. (43) also demonstrated an independent association between TyG-WC and hypertension and cardiovascular risk. Research has shown that TyG-WC has better predictive performance for newly developed CVD than TyG and TyG-BMI (44). Alternative indicators of IR may help predict the occurrence of cardiovascular diseases, especially hypertension.

This study utilized data from a community-based population in a cross-sectional study to investigate the relationship between the TyG-WC index and H-type hypertension. Our findings suggest that the TyG-WC index is a better associated factor for the risk of H-type hypertension than the TyG index alone. As WC increases, it may be accompanied by visceral fat accumulation, metabolic syndrome, insulin resistance, elevated blood pressure, and sympathetic nervous system activation, which can be explained by the close interplay between blood pressure, blood glucose, and triglycerides. Compared to BMI, WC appears to have a stronger association with central arterial stiffness and dysfunctional adipose tissue, both of which are prone to increasing the risk of hypertension (45, 46). The TyG index may assess hypertension through the level of IR, and IR can influence hypertension by activating the sympathetic nervous system. IR-induced insulin abnormalities lead to excessive activation of the sympathetic nervous system. Enhanced sympathetic nervous system activity can cause vasoconstriction, increased cardiac output, and fluid retention, thereby elevating blood pressure (47). This effect is particularly pronounced in H-type hypertension. In our study, we found that TyG-WC, a combination of abdominal obesity and TyG, is a biomarker for predicting HTH. The superiority of TyG-WC may be due to the well validated roles of TG, FPG, and obesity status in the occurrence and development of IR and HTH. Future larger prospective studies are needed to further investigate the association between the TyG-WC index and H-type hypertension across different populations and subgroups.

This study we conducted has some strengths. First, to our knowledge, this is the first study to explore the TyG-WC index serves as an independent risk factor for HTH, potentially contributing to its tertiary prevention strategies. Next, TyG-WC index are not only easy to obtain, but also have good predictive performance, simple and practical. Meanwhile, this study provides evidence for comparing the discriminative efficacy between TyG-WC and traditional indicators (such as HOMA-IR). And then our study, confirmed the independent relationship between TyG-WC and HTH. ROC curve analysis of multiple continuous variables indicates that TyG-WC is the strongest independent predictor of HTH. Ultimately, the comparative evaluation of TyG-WC's discriminatory performance in distinguishing HTH from general hypertension within a population-based setting revealed its superior suitability for HTH identification relative to conventional hypertension. This study establishes a simple, cost-effective screening tool for metabolic assessment of HTH and may provide novel perspectives for future evaluation of potential HTH patients. Through these reliable statistical analyses, we can conclude that the results of this study are relatively true and reliable, and can be extended to a considerable portion of the community population.

However, some limitations should be aware when interpreting the results of the study. Primarily, as a cross-sectional investigation, it possesses limited capacity to establish causal relationships.Cross-sectional studies primarily identify associations rather than establish definitive predictive causality. In our analysis, TyG-WC showed a statistically significant discriminative capacity (AUC = 0.676, *P* = 0.010) compared to other variables, suggesting its potential as a biomarker. Future cohort studies are warranted to validate the predictive performance of the TyG-WC index, particularly through refined subgroup analyses in Pre-diabetic populations and Diverse hypertension subtypes. Secondly, the study relies on data and indicators from a single historical time point, with TyG-WC represented as a static baseline value. This approach precludes the assessment of temporal variations and cumulative effects of TyG-WC, potentially leading to an underestimation of its association with the disease under investigation. Finally, the scope of our research is confined to selected urban and rural communities within six cities in Hunan, China. The restricted sample size and geographical limitations constrain the generalizability of our findings to other regions. Consequently, further research is imperative to validate the applicability of our principal findings across diverse geographical locations and ethnic populations.

## 5 Conclusion

In this study, we demonstrated that the TyG-WC index serves as an independent risk factor for HTH, potentially contributing to its tertiary prevention strategies. The TyG-WC index emerged as a valuable predictive marker for HTH due to its strong association with disease occurrence. Notably, its calculation from routine laboratory parameters facilitates practical application, suggesting its potential utility in risk assessment during epidemiological surveys.

## Data Availability

The data that support the findings of this study are available from the corresponding author upon reasonable request.
