# Supplementary material for: Association between triglyceride-glucose index multiplied by waist circumference and H-type hypertension among Chinese adults
Source: Front Cardiovasc Med. 2025 Jul 10;12:1589488. doi: 10.3389/fcvm.2025.1589488 (PMC12287031; doi:10.3389/fcvm.2025.1589488)
Supplement: Supplementary file 1 [file Table1.docx]

**Table S1** Baseline characteristics of community residents according to health status (single-HHcy, single-hypertension and HTH).

| **Characteristics** | **HTH** | **Single-Hypertension** | **Single-HHcy** | **Healthy group** | ***P value*** |
| --- | --- | --- | --- | --- | --- |
| Sex, n (%), |  |  |  |  |  |
| male | 444 (56.0) | 262 (43.6) | 302 (48.2) | 636 (31.9) | ＜0.001 |
| Female | 349 (44) | 339 (56.4) | 325 (51.8) | 1 355 (68.1) |  |
| Smoking,n (%) |  |  |  |  | ＜0.001 |
| Yes | 249 (31.4) | 109 (18.1) | 148 (23.6) | 336 (16.9) |  |
| No | 544 (68.6) | 492 (81.9) | 479 (76.4) | 1655 (83.1) |  |
| Drinking, n (%) |  |  |  |  | 0.008 |
| Yes | 236 (29.8) | 119 (19.8) | 188 (30) | 420 (21.1) |  |
| No | 557 (70.2) | 482 (80.2) | 439 (70) | 1571 (78.9) |  |
| Age (years) | 59.6 (12.7) | 56.9 (10.8) | 52.1 (12.6) | 52.7 (12.4) | ＜0.001 |
| WC (cm) | 88.3 (10.5) | 87.5 (9.3) | 83.9 (9.4) | 82.0 (9.2) | ＜0.001 |
| SBP (mmHg) | 149.1 (18.7) | 149.1 (15.3) | 116.1 (11.3) | 115.7 (11.3) | ＜0.001 |
| DBP (mmHg) | 91.0 (11.4) | 89.3 (10.4) | 74.7 (7.1) | 73.7 (8.1) | ＜0.001 |
| TG (mmol·L-1) | 2.5 (2.1) | 1.9 (1.6) | 2.4 (1.7) | 1.7 (1.3) | ＜0.001 |
| TC (mmol·L-1) | 5.0 (1.0) | 4.8 (0.9) | 5.2 (1.0) | 4.7 (0.9) | ＜0.001 |
| FBG (mmol·L-1) | 6.0 (1.5) | 5.6 (1.4) | 5.5 (1.1) | 5.6 (1.7) | ＜0.001 |
| HDL-C (mmol·L-1) | 1.2 (0.3) | 1.3 (0.3) | 1.3 (0.3) | 1.4 (0.3) | ＜0.001 |
| LDL-C (mmol·L-1) | 2.6 (0.9) | 2.7 (0.8) | 2.8 (1.0) | 2.6 (0.8) | ＜0.001 |

**Table S2** Association between TyG-WC index and two types of hypertension in different models.

| **Characteristics** | **Model 1** |  | **Model 2** |  | **Model 3** |  |
| --- | --- | --- | --- | --- | --- | --- |
|  | **OR (95%CI)** | ***P valve*** | **OR (95%CI)** | ***P valve*** | **OR (95%CI)** | ***P valve*** |
| TyG-WC1 | 1.005  (1.004~1.006) | <0.001 | 1.004  (1.003 ~ 1.005) | <0.001 | 1.004  (1.001 ~ 1.007) | <0.001 |
| TyG-WC 2 | 1.007 | <0.001 | 1.006 | <0.001 | 1.006 | <0.001 |
|  | (1.006~1.008) |  | (1.006~1.007) |  | (1.005~1.007) |  |
| TyG-WC (%) 1 | 1.570  (1.443 ~ 1.709) | <0.001 | 1.485  (1.359 ~ 1.622) | <0.001 | 1.342  (1.047 ~ 1.720) | 0.020 |
| TyG-WC (%) 2 | 1.986  (1.831 ~ 2.153) | <0.001 | 1.856  (1.703 ~ 2.204) | <0.001 | 1.696  (1.532 ~ 1.877) | <0.001 |
| Model 1: unadjusted;  Model 2: Adjust age and gender;  Model 3: Adjust for age, gender, smoking, alcohol consumption, triglycerides, total cholesterol, high-density lipoprotein, low-density lipoprotein, and fasting blood glucose. | | | | | | |

TyG-WC1 shows the association between continuous variable TyG-WC and general hypertension in different models, with the control group being a healthy population;

TyG-WC2 shows the association between continuous variable TyG-WC and HTH in different models, with the control group being a healthy population;

TyG-WC (%)1 shows the association between TyG-WC quartile and general hypertension in different models, with the control group being a healthy population;

TyG-WC (%)2 shows the association between TyG-WC quartile and HTH in different models, with the control group being a healthy population.


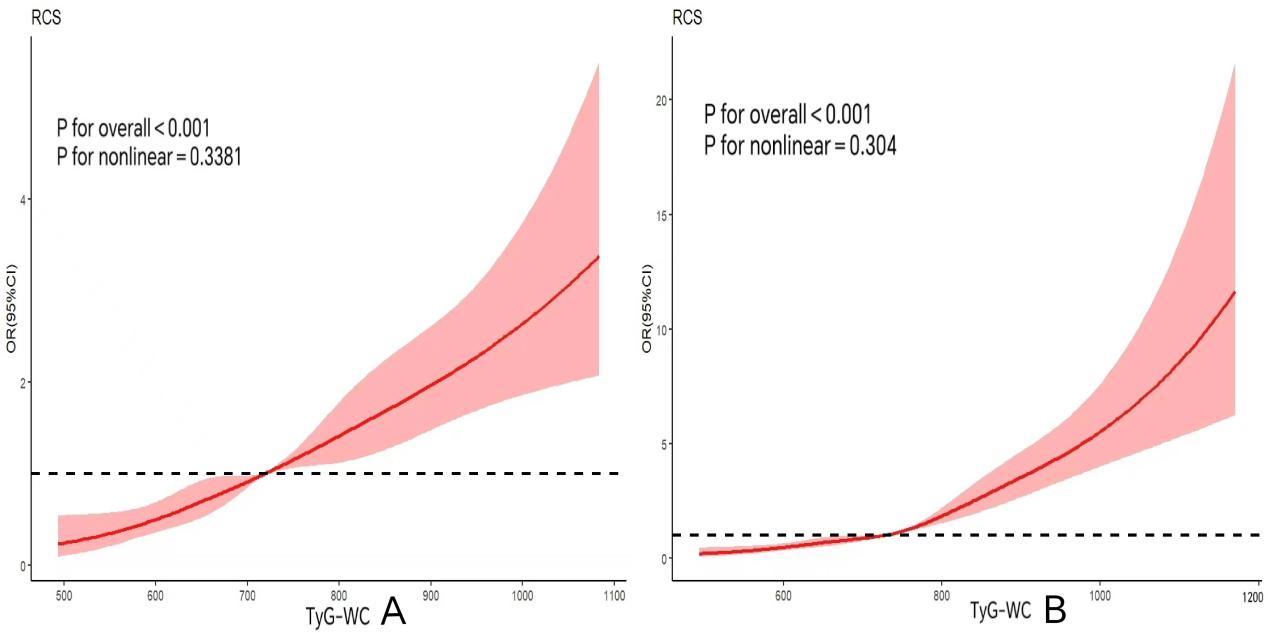


**Figure S1** RCS curves of TyG-WC and two types of hypertension.

Figure A shows the RCS curves of the relationship between TyG-WC index and general hypertension, with the control group being a healthy population. Figure B shows the RCS curves of the relationship between TyG-WC index and HTH with the control group being a healthy population.

The variables for age, and gender were all modified.


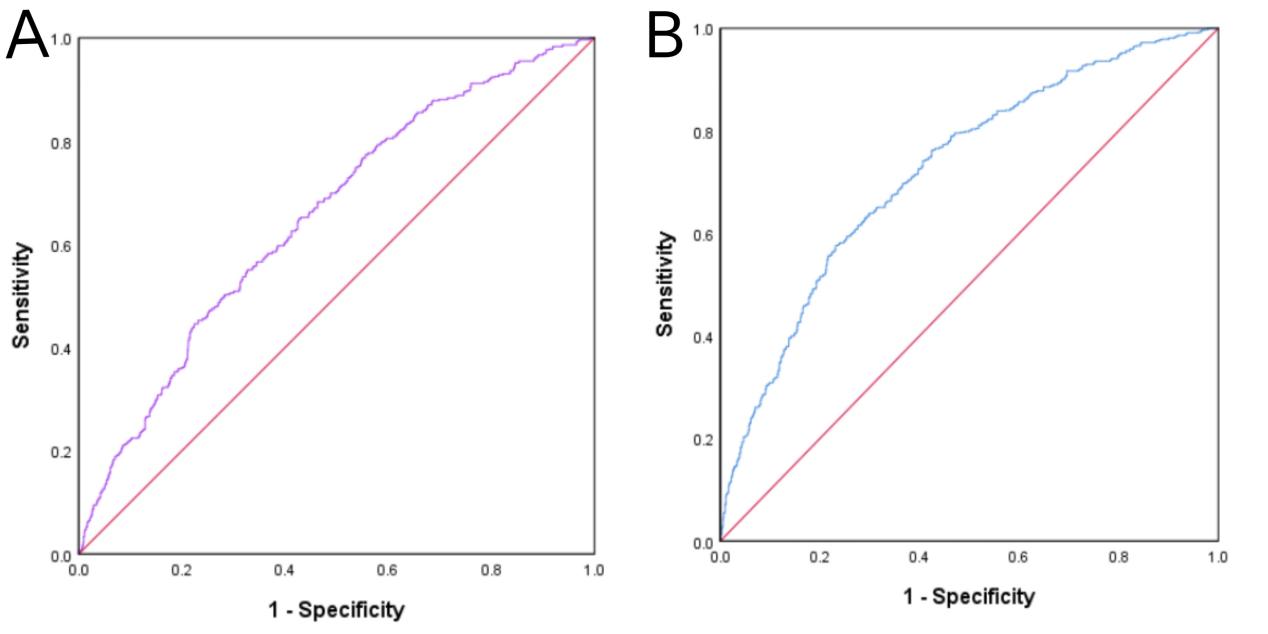


**Figure S2** ROC curves of TyG-WC and two types of hypertension

Figure A shows the ROC curves of the relationship between TyG-WC index and general hypertension, with the control group being a healthy population. Figure B shows the ROC curves of the relationship between TyG-WC index and HTH with the control group being a healthy population.
